# Adolescent Exposure to Nicotine and Ethanol Attenuates Adult Nicotine Reward Sensitivity in Rats

**DOI:** 10.1002/npr2.70132

**Published:** 2026-05-20

**Authors:** Michimasa Toyoshima, Ryota Toyofuku, Shota Shimoda, Lena Tsuchida, Reina Tachihara, Kazuo Yamada

**Affiliations:** ^1^ Laboratory of Psychology and Behavioral Neuroscience, Institute of Human Sciences University of Tsukuba Tsukuba Ibaraki Japan

**Keywords:** adolescence, conditioned place preference, co‐use, ethanol, nicotine, rats, reward

## Abstract

Adolescence is a sensitive developmental stage in which exposure to addictive substances can produce long‐lasting changes in the neural circuits that regulate reward. Nicotine and ethanol are frequently co‐used among adolescents, and converging evidence suggests that these drugs interact to modulate mesolimbic dopamine transmission. The present study investigated how chronic exposure to nicotine, ethanol, or their combination during adolescence influences adult conditioned place preference (CPP) for nicotine. Male Wistar‐Imamichi rats were assigned to four groups: Control (no drug exposure), Nicotine (0.20 mg/kg/day, s.c., PND 28–41), EtOH (voluntary 20% ethanol intake), and Co‐use (nicotine + ethanol). After maturation, all animals underwent CPP with 0.08 mg/kg nicotine using an unbiased procedure. We hypothesized that adolescent drug exposure would enhance vulnerability to nicotine reward in adulthood. Contrary to this hypothesis, only the Control group exhibited significant CPP, whereas no nicotine‐induced preference was observed in any drug‐exposed group. These findings emphasize the complexity of developmental drug interactions and suggest that adolescent exposure to addictive substances does not uniformly increase vulnerability to nicotine reward.

## Abstract 2

We investigated how chronic exposure to nicotine, ethanol, or their combination during adolescence influences adult conditioned place preference (CPP) for nicotine. No significant CPP was observed in any of the drug‐exposed groups, suggesting adolescent exposure to addictive substances does not uniformly increase vulnerability to nicotine reward.

## Graphical abstract

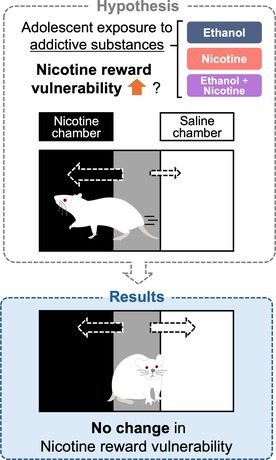

## 1 Introduction

Nicotine dependence typically emerges during adolescence, a developmental stage characterized by marked remodeling of cortical and subcortical circuits that govern reward processing, executive function, and emotional regulation [1]. According to the National Survey on Drug Use and Health, approximately 90% of adult smokers initiate cigarette use before the age of 18 [2], suggesting that nicotine exposure during adolescence may exert particularly strong and long‐lasting effects on later vulnerability to dependence. During this period, nicotinic acetylcholine receptors (nAChRs) and dopaminergic projections from the ventral tegmental area (VTA) undergo substantial refinement, rendering the adolescent brain especially susceptible to pharmacological perturbation [1, 3, 4].

Ethanol exposure frequently accompanies nicotine use in adolescents. Human studies show that alcohol consumption increases craving for cigarettes and enhances subjective nicotine reward [5], whereas preclinical studies have demonstrated synergistic effects of nicotine and ethanol on VTA dopaminergic activity [6]. These findings raise the possibility that adolescents who engage in combined nicotine and alcohol use may undergo neurobiological changes that heighten susceptibility to later addiction.

However, empirical evidence on the long‐term behavioral effects of adolescent co‐exposure remains limited. Some studies indicate that adolescent ethanol exposure can decrease sensitivity to certain effects of nicotine in adulthood, including its reinforcing properties [7, 8, 9], whereas others have demonstrated enhanced nicotine‐related responses, particularly in mesolimbic dopaminergic activity, depending on the dose and pattern of alcohol administration [10, 11, 12]. Moreover, few investigations have directly compared nicotine‐only, ethanol‐only, and combined exposure within the same developmental window. Understanding these interactions is essential for clarifying addiction mechanisms and informing prevention strategies targeting adolescent substance use.

In the present study, we examined whether chronic exposure to nicotine, ethanol, or both during adolescence alters adult sensitivity to nicotine reward. We assessed conditioned place preference (CPP) induced by low‐dose nicotine, allowing detection of enhanced reward sensitivity. We hypothesized that adolescent drug exposure would increase vulnerability to nicotine reward. The results, however, did not support this expectation.

## 2 Materials and Methods

### 2.1 Animals

A total of 44 male Wistar‐Imamichi rats were used. Ten rats were assigned to a preliminary dose–response study and 34 to the main experiment. Animals were housed individually under a 12:12 light–dark cycle (lights on at 08:00) with food and water available ad libitum. All experiments were carried out according to the guidelines for the Care and Use of Animals approved by the University of Tsukuba Committee on Animal Research.

### 2.2 Preliminary Nicotine Dose–Response Study

Rats received nicotine injections (0.04, 0.08, or 0.12 mg/kg, s.c.) paired with one compartment of a three‐chamber CPP apparatus using an unbiased design. Baseline preferences were assessed over two sessions; conditioning was conducted over 6 days; CPP was measured on the following day. The purpose was to identify a nicotine dose that produces only minimal CPP, allowing enhancement to be detected.

### 2.3 Adolescent Drug Exposure

Beginning on PND 28, rats were randomly assigned to one of four groups: Control (saline only), Nicotine (nicotine injections), EtOH (voluntary ethanol intake), or Co‐use (combined nicotine and ethanol exposure). Nicotine (0.20 mg/mL, pH 7.0) was administered at 1 mL/kg/day for 14 days. This nicotine dose was selected because once‐daily subcutaneous injections of 0.20 mg/kg are known to be sufficient to induce neuroplastic changes in the adolescent rodent brain without causing overt toxicity [13, 14]. Ethanol exposure consisted of continuous two‐bottle choice between water and 20% ethanol; intake was measured every 2 days. The 20% ethanol concentration was chosen to promote high levels of intake that model human heavy drinking patterns during adolescence [15]. By using these doses, we aimed to test whether chronic exposure to levels of these drugs that are significant but do not necessarily induce immediate motor or physiological impairment would sensitize the reward system to subsequent nicotine exposure in adulthood.

### 2.4 Adult Nicotine Conditioned Place Preference

From PND 70, rats underwent CPP testing with 0.08 mg/kg nicotine. The procedure consisted of habituation, two baseline sessions, six conditioning sessions with alternating nicotine and saline injections, and a final test. Behavior was recorded and analyzed using Any‐maze software.

### 2.5 Statistical Analysis

CPP scores in each group were calculated as the time spent in the nicotine‐paired chamber during the Test session minus the time spent during the Baseline session. Data were compared to the chance level, which indicates that rats spent an equal amount of time in the nicotine‐ and saline‐paired chambers using one‐sample *t*‐test. Ethanol consumption and ethanol preference ratio (ethanol intake relative to total fluid intake) were analyzed using a two‐way ANOVA (Phase × Group), followed by repeated‐measures ANOVA within each group. Statistical significance was set at *p* < 0.05.

## 3 Results

### 3.1 Preliminary Study

**FIGURE 1:**
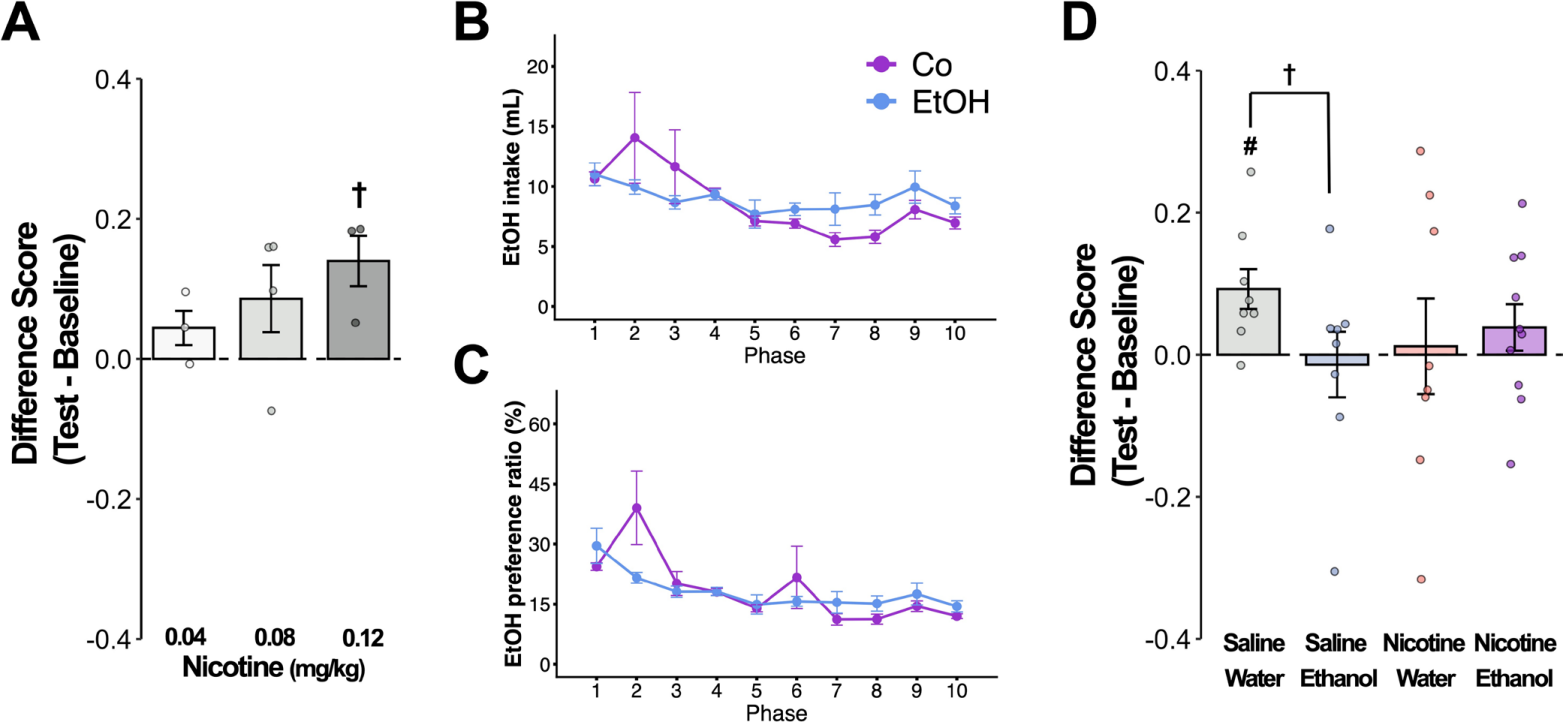
Effects of adolescent nicotine and ethanol exposure on adult nicotine reward sensitivity. (A) Preliminary dose–response study for nicotine‐induced CPP. Difference scores (time in nicotine‐paired chamber minus time in saline‐paired chamber) are shown for three doses: 0.04, 0.08, and 0.12 mg/kg (*n* = 3–4 per dose). (B) Adolescent voluntary ethanol intake. Daily 20% ethanol consumption in the EtOH and Co‐use groups from PND 28 to 41. Intake significantly declined over the 10‐phase exposure period (*p* < 0.05). (C) Adolescent EtOH preference ratio. EtOH preference ratio (EtOH intake relative to total fluid intake) showed a non‐significant decrease from approximately 30% on PND 28% to 15% on PND 41. (D) Adult nicotine CPP scores following adolescent drug exposure. CPP was tested with 0.08 mg/kg nicotine in four groups: Control (Saline/Water), Nicotine (Nicotine/Water), EtOH (Saline/Ethanol), and Co‐use (Nicotine/Ethanol) (*n* = 8–9 per group). Only the Control group exhibited significant preference (*p* < 0.05 vs. chance level). †: *p*< 0.01, #: *p*> 0.05 vs. chance level.

Only the 0.12 mg/kg nicotine group showed a trend toward increased preference (*t* (2) = 3.17, *p* < 0.10), whereas the 0.04 and 0.08 mg/kg doses produced no significant CPP (Figure 1A). Based on these findings, 0.08 mg/kg was selected as the sub‐threshold dose for the main experiment.

### 3.2 Adolescent Ethanol Intake

Ethanol consumption in the EtOH and Co‐use groups declined gradually across the exposure period, despite growth‐associated increases in total fluid intake (*F* (9,144) = 3.29, *p* < 0.05) (Figure 1B). Concurrently, the ethanol preference ratio decreased from approximately 30% on PND 28 to 15% on PND 41, although this change did not reach statistical significance (Figure 1C).

### 3.3 Adult Nicotine CPP

One‐sample *t*‐test revealed that only the Control group exhibited a significant preference for the nicotine‐paired chamber (*t* (7) = 3.08, *p* < 0.05), whereas none of the drug‐exposed groups showed significant CPP (Figure 1D). Furthermore, inter‐group comparisons indicated that the CPP score in the Control group tended to be higher than that in the EtOH group (*p* < 0.10) (Figure 1D) (see Supporting Information S1: Raw Data (Nicotine_Ethanol_CPP).xlsx).

## 4 Discussion

The present study examined whether adolescent exposure to nicotine, ethanol, or their combination would alter adult sensitivity to nicotine reward. Contrary to our hypothesis, none of the drug‐exposed groups demonstrated enhanced CPP to a low dose of nicotine in adulthood. These results are consistent with findings reported by Boutros et al. (2016) [7], who showed that adolescent intermittent ethanol exposure attenuated, rather than enhanced, nicotine‐induced CPP in adulthood. Their study similarly indicated that prior ethanol exposure blunted the rewarding effects of nicotine, suggesting that certain patterns of adolescent drug exposure may reduce, rather than increase, sensitivity to nicotine reward later in life.

In interpreting these findings, it is important to consider the drug‐exposure procedures used during adolescence. Nicotine was administered passively via subcutaneous injections at body‐weight–adjusted doses, whereas ethanol was consumed voluntarily through a two‐bottle choice procedure, making precise control of ethanol intake impossible. Although ethanol consumption gradually declined over days—resulting in a decreasing proportion of ethanol relative to total fluid intake—the absolute amount consumed remained sufficiently high to exert meaningful neurobiological effects on the adolescent brain. In the present study, rats consumed an average of 16.70 g of ethanol between PND 28 and 48, with daily intake of 4.23 g/kg. For comparison, when extrapolated to a 15‐year‐old human male (approx. 59.2 kg), this dose corresponds to approximately 250 g of pure ethanol per day—equivalent to more than 12 “go” (2.16 L) of 15% sake. Given that the Japanese Ministry of Health, Labour and Welfare defines heavy drinking as exceeding 60 g of pure ethanol per day [16], the ethanol exposure in our model was clinically substantial.

On the other hand, the drug‐exposure regimen may not have been sufficient to induce long‐term neurobehavioral modification. Although both nicotine and ethanol doses were comparable to those used in previous developmental studies, subtle differences in duration, route of administration, or peak blood concentration may influence susceptibility to persistent alterations in reward circuitry [13, 17, 18]. From a neuropharmacological perspective, the lack of enhanced CPP in the co‐exposed groups may be attributed to complex modulations within the mesolimbic dopamine system. Adolescent drug exposure can alter the expression of nAChRs, particularly the α4β2 and α7 subtypes, in the VTA and nucleus accumbens [1, 10]. It is possible that our adolescent exposure regimen led to a long‐term desensitization or down‐regulation of these receptors rather than sensitization. Furthermore, chronic nicotine and ethanol may differentially impact tonic and phasic dopamine release; while acute exposure typically enhances phasic firing of VTA neurons, chronic developmental exposure can “freeze” the reward circuit in an immature state or alter the balance of GABAergic and glutamatergic inputs to the VTA [4, 17], thereby blunting the incentive salience of subsequent nicotine. The absence of cross‐sensitization in this study suggests that the interaction between nicotine and ethanol during adolescence may induce compensatory adaptations that mask or counteract any potential increase in reward sensitivity.

Furthermore, continuous or higher‐dose nicotine exposure, particularly via osmotic minipumps, has been shown to induce stronger and longer‐lasting neuroplastic changes in adolescent rodents than once‐daily injections [13, 14]. Similarly, ethanol exposure patterns that produce repeated cycles of intoxication and withdrawal, or that generate higher peak blood ethanol concentrations, may yield more robust behavioral and neural effects [17, 18, 19].

In addition, the CPP paradigm itself may have limited sensitivity to nicotine reward. Nicotine generally produces modest conditioned reinforcement relative to psychostimulants [20], and the unbiased CPP design is known to further reduce sensitivity to nicotine‐induced place preference [20, 21]. Some studies have reported nicotine CPP only under biased procedures or when environmental stimuli strongly interact with drug effects [22]. It is therefore plausible that adolescent exposure altered subtle aspects of nicotine reward processing—such as incentive salience, motivational vigor, or reinforcement efficacy—that might be more readily detected using intravenous self‐administration procedures [23] rather than CPP.

It is also important to note that the interaction between nicotine and ethanol during adolescence may not uniformly increase vulnerability to nicotine reward. Human studies indicate notable heterogeneity: while co‐use is associated with heavier consumption and greater dependence in many adolescents [24], other evidence suggests reduced sensitivity to nicotine reinforcement following heavy alcohol involvement. Such variability may reflect differences in genetic background, stress exposure, developmental timing, or drug‐use patterns—factors that could similarly modulate outcomes in rodent models [17, 25].

Overall, the present findings contribute to a nuanced understanding of developmental drug exposure by demonstrating that co‐exposure to nicotine and ethanol does not inevitably enhance susceptibility to nicotine reward. Future studies should incorporate broader dose–response assessments, biased CPP procedures, self‐administration paradigms, and more intensive drug‐exposure regimens to fully characterize potential interaction effects.

## Author Contributions

Ryota Toyofuku, Shota Shimoda, and Kazuo Yamada conceptualized and designed experiments. Ryota Toyofuku and Lena Tsuchida performed the behavioral tests. Michimasa Toyoshima, Reina Tachihara, and Kazuo Yamada analyzed the data and wrote the manuscript. Kazuo Yamada supervised all aspects of the present study. All authors have read and approved the final manuscript.

## Funding

This work was supported by the Japan Society for the Promotion of Science (JP 19K21806 to K.Y.).

## Ethics Statement

Animal experiments were approved by the University of Tsukuba Committee on Animal Research.

## Conflicts of Interest

The authors declare no conflicts of interest.

## Supporting information


**Data S1:** npr270132‐sup‐0001‐DataS1.xlsx.

## Data Availability

The data that supports the findings of this study are available in the Supporting Information S1 of this article.
